# A Comparative Analysis of Skeletal and Dental Parameters in Bilateral Cleft Lip and Palate vs. Non-bilateral Cleft Lip and Palate Patients in the Central Indian Population: A NemoCeph Study

**DOI:** 10.7759/cureus.54497

**Published:** 2024-02-19

**Authors:** Shivani S Rawat, Vikrant V Jadhav, Priyanka Paul

**Affiliations:** 1 Public Health Dentistry, Sharad Pawar Dental College and Hospital, Datta Meghe Institute of Higher Education and Research, Wardha, IND; 2 Orthodontics and Dentofacial Orthopaedics, Sharad Pawar Dental College and Hospital, Datta Meghe Institute of Higher Education and Research, Wardha, IND

**Keywords:** cleft lip and palate, point a, orthognathic surgery, nemoceph, tweed’s analysis, steiner’s analysis, down’s analysis, cephalometric analysis

## Abstract

Introduction

Orthodontic diagnosis and treatment planning encounter distinctive complexities when dealing with cleft lip and palate anomalies. This research endeavors to thoroughly examine skeletal and dental characteristics through cephalometric analyses among individuals with bilateral cleft lip and palate (BCLP) within the central Indian population. Due to anatomical variations and growth constraints, traditional cephalometric mean values derived from standard population studies are often inadequate for these cases. Advanced technology, such as NemoCeph (Nemotech, Madrid, Spain) software, enhances measurement accuracy.

Methods

Fifty patients, including 25 with BCLP and 25 without BCLP, aged 10 to 18, were selected for this cross-sectional study. Lateral cephalograms were traced and analyzed using NemoCeph software. Skeletal and dental parameters were measured, and a comparison was made between BCLP patients and the general population. Statistical analysis was conducted using the Student’s unpaired t-test. Both SPSS Statistics Version 24.0 (IBM Corp., Armonk, NY, USA) and GraphPad Prism Version 7.0 (GraphPad Software, San Diego, CA, USA) were used for data analysis.

Results

The investigation revealed significant disparities across several parameters, including sella-nasion-A point angle (SNA), sella-nasion-B point angle (SNB), A point-nasion-B point angle (ANB), the inter-incisal angle (the angle between the long axes of the upper and lower incisors), and UP 1 to A-pog (a specific vertical measurement between anatomical markers labeled “upper 1” and “A point to pogonion”), with associated p-values for the skeletal and dental parameters of 0.310, 0.259, 0.195, 0.0001, and 0.0001, respectively. A comparison between manual tracing and digital methods indicated a reduction in errors and an improvement in measurement precision. Notably, patients diagnosed with BCLP exhibited distinctive skeletal and dental traits, highlighting the necessity for tailored treatment approaches.

Conclusion

This study emphasizes the importance of personalized cephalometric evaluations for patients with BCLP. Standard mean values may not be applicable due to unique anatomical considerations in these cases. Advanced technology and patient-specific assessments are crucial for accurate diagnosis, treatment planning, and orthognathic procedures in individuals with cleft lip and palate conditions. Embracing digital tools and tailored approaches can enhance patient care quality and lead to better clinical outcomes.

## Introduction

Orthodontics represents a continuous and phased approach to treatment, as emphasized by Mayne [1]. This discipline encompasses the comprehensive assessment of craniofacial and bodily growth and development, with a particular emphasis on their influence on dental alignment. It delves into understanding the interplay between intrinsic and extrinsic factors that shape growth patterns, as well as the identification and correction of any deviations or impediments [2]. In the diagnostic process, such as during the evaluation of cephalometric radiographs, experts may require more detailed information. For a layperson, a common question arises: "What kind of insights can be derived from lateral or frontal cephalometric head films?" Cephalometry, utilizing radiographic techniques, has been an integral component of orthodontics for over half a century. The acquisition, tracing, and interpretation of cephalometric radiographs are fundamental procedures in orthodontic practice. In contrast to traditional film-based radiography, direct digital systems employ charge-coupled detectors or storage phosphor plates, offering numerous advantages over traditional radiographic films. These advantages encompass reduced radiation exposure for patients, immediate generation of radiographic images, elimination of the need for darkrooms, associated costs and time for development, streamlined image enhancement, storage, and management, simplified sharing of images with relevant professionals, and automated landmark identification [3,4]. A plethora of commercial software options, including Quick Ceph, Dolphin, NemoCeph (Nemotech, Madrid Spain), Vistadent, TraceCeph, CephX, and Facad, are available to facilitate the execution of cephalometric analysis.

The recent development in the field of digital technology replaced the manual tracing method with semiautomated computer-based software, such as NemoCeph, which enables direct soft tissue and hard tissue landmark identification on-screen displayed digital images [5]. NemoCeph offers an extensive range of capabilities for cephalometric analysis, addressing both lateral and frontal perspectives. Its customization tools empower users to craft personalized cephalometric analyses. An outstanding feature is its remarkable precision in the localization of cephalometric points, made possible by a diverse set of basic and advanced image processing tools. The software also allows for the concurrent tracing of multiple analyses, enabling on-the-fly adjustments. NemoCeph encompasses growth prediction tracings and the ability to convert cephalometric data, including condylar centric relation, visual treatment objective (VTO), and surgical treatment objective (STO). What sets it apart is its seamless integration of cephalometric VTO with dental VTO, enabling predictions that account for factors like profile analysis and space considerations. These predictions aid in evaluating the potential for various procedures such as extractions, stripping, and expansions. Significantly, the STO functionality is invaluable for making predictions related to surgical, pre-surgical, and pre-orthodontic scenarios, covering a broad spectrum of osteotomies, including bilateral sagittal split osteotomy (BSSO), vertical mandibular, maxillary, occlusal plane alterations, seminary, and genioplasty. This comprehensive scope offers precise control over the selected osteotomies. Additionally, NemoCeph supports morphing in both lateral and frontal views, enhancing its analytical capabilities while maintaining originality [6]. The utilization of such software significantly aids orthodontic practitioners in conducting cephalometric assessments and devising precise diagnostic and treatment strategies.

The utilization of digital cephalometrics in orthodontic practices is on the rise, with the capability of transferring images directly to a computer database, as noted by Bruntz et al. They found that when converting analog film to digital format using a scanner, there were instances of both vertical and horizontal distortion [7]. A software application employing vector algebra has been developed to facilitate the precise measurement of cephalometric angles and distances [8,9].

In orthodontics, Steiner’s and Downs’ analyses are commonly used, while Tweed’s analysis is less prevalent. Downs’ analysis illustrates typical dental and facial patterns, serving as a starting point for treatment planning. Steiner's analysis, established in 1953, is widely used, offering a comprehensive assessment of skeletal, dental, and soft tissue aspects. It evaluates occlusal and mandibular planes, incisor positions, and relationships between skeletal components. Tweed's analysis focuses on lower incisor positioning relative to facial structures and basal bone [10-12]. Cleft lip and palate (CLP) represent a congenital craniofacial anomaly, ranking as the second most common structural birth deformity after clubfoot. This condition is known to result from various factors, including maternal smoking and alcohol consumption during pregnancy. It can give rise to challenges such as feeding difficulties, speech impairments, hearing problems, and recurrent ear infections [13,14]. Patients with CLP often have distorted maxillary structures and craniofacial deformities, making it challenging to locate specific anatomical points on cephalometric radiographs. This difficulty reduces data accuracy, particularly in identifying pairs of points like gonion and orbitale and individual landmarks such as A point or those related to maxillary incisors. Cephalometric studies are typically not conducted due to hypoplastic maxillae and impaired growth in these patients.

Furthermore, the primary focus of this study is to determine the accuracy and consistency of Downs’, Steiner's, and Tweed's cephalometric analyses as applied to individuals within the central Indian population who are affected by bilateral cleft lip and palate (BCLP). In addition to the cephalometric assessments, this research endeavor aims to provide a comprehensive evaluation of both the skeletal and dental characteristics exhibited by individuals with BCLP. Moreover, the study is designed to facilitate comparative analysis, enabling a thorough examination of the distinctions in skeletal and dental traits between individuals diagnosed with BCLP and those who do not present with this condition.

This study addresses the notable absence of cephalometric analyses tailored to patients in need of such assessments [15]. Through the findings of this research, we aim to accurately locate A point and other critical landmarks in patients with CLP, ultimately contributing to the development of standardized treatment plans, the execution of orthognathic procedures, and the implementation of a low-radiation analytical approach, thereby addressing a critical clinical need in this patient population.

## Materials and methods

The objective of this study is to investigate craniofacial characteristics in unoperated BCLP individuals compared to a control group without BCLP, focusing on ages 10 to 18.

Participants

BCLP Group

The BCLP group comprises 25 individuals with unoperated BCLP.

Control Group

The control group consists of 25 individuals without BCLP, who were previously treated with nasoalveolar molding surgery at one year old.

Ethical considerations

Informed Consent

Informed consent was obtained through written consent from all participants or their guardians.

Ethics Approval

Ethics approval was granted by the Datta Meghe Institute of Higher Education and Research (DMIHER(DU)/IEC/2023/705) ethics committee.

Selection criteria

Inclusion

Selection criteria included individuals aged 10 to 18 with class I or III skeletal categories and maxillary deficiency.

Exclusion

Exclusion criteria comprised individuals who had prior orthodontic treatment for CLP or syndromic conditions to avoid confounding variables.

Data collection

Imaging

Data collection involved the acquisition of intraoral photographs and lateral cephalograms before orthodontic treatment, ensuring standardized capture conditions for imaging.

Radiographs

Radiographs were obtained in centric occlusion to maintain relaxed lips and standard head positioning.

Clinical examination

The assessment included case history, impression taking, model analysis, and classification based on Angle’s malocclusion.

Cephalometric analysis

Software

NemoCeph was used for measuring cephalometric parameters and comparing individuals with BCLP to the general population.

Landmark Utilization

Nasion was consistently employed as a reference point to establish A point.

Methods

**Figure 1:**
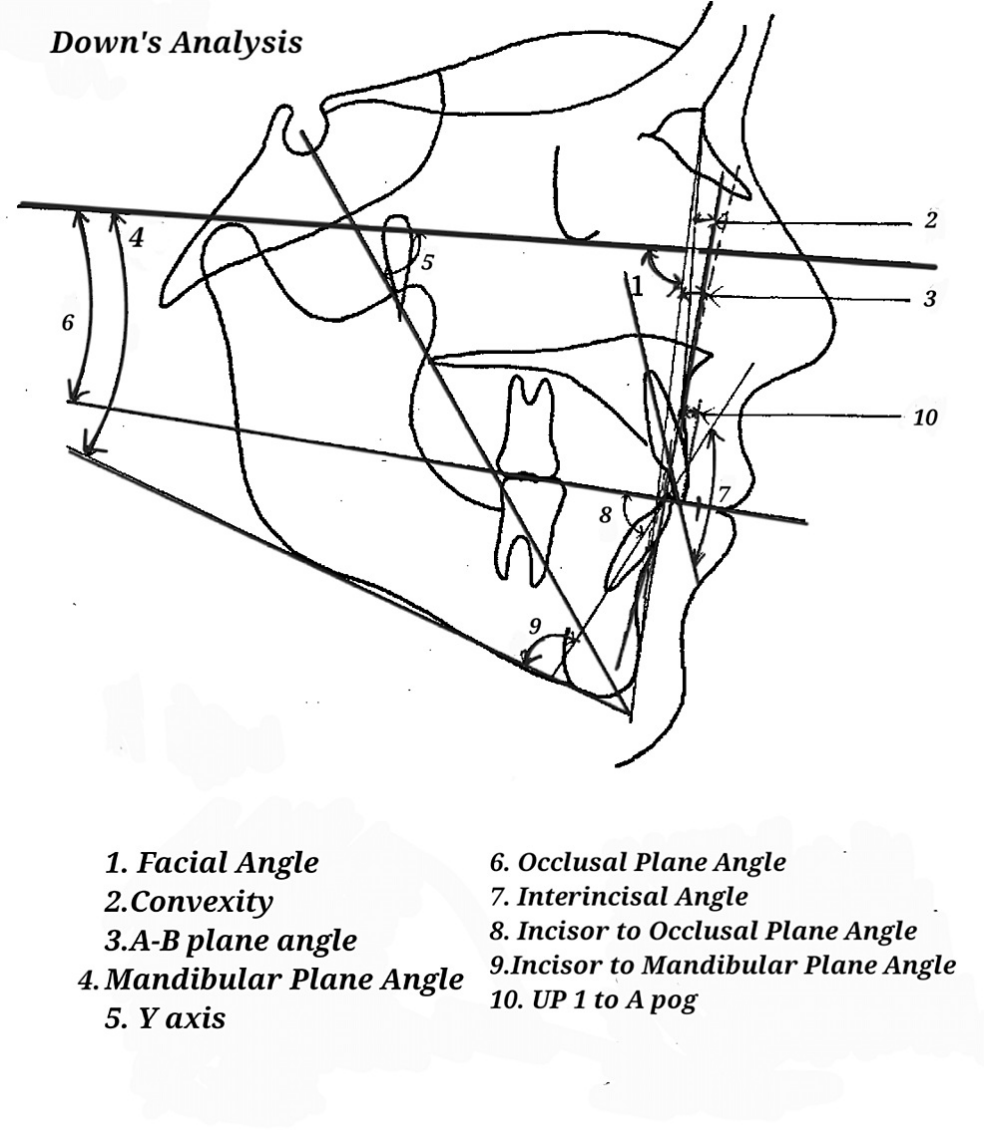
Downs’ analysis. Image has been created by the authors of this article.

**Figure 2:**
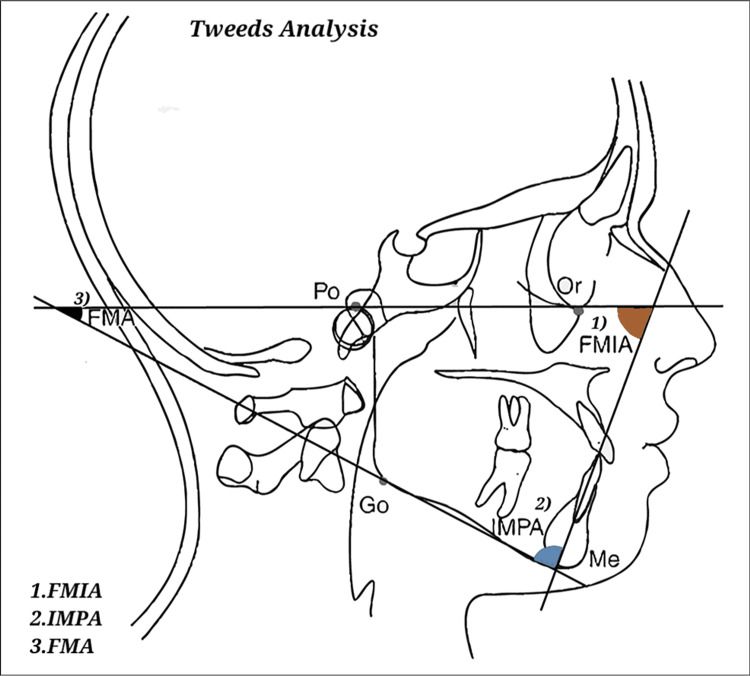
Steiner's analysis. Image has been created by the authors of this article. FMA, Frankfort-mandibular plane angle; FMIA, Frankfort-mandibular incisor angle; IMPA, incisor mandibular plane angle

**Figure 3:**
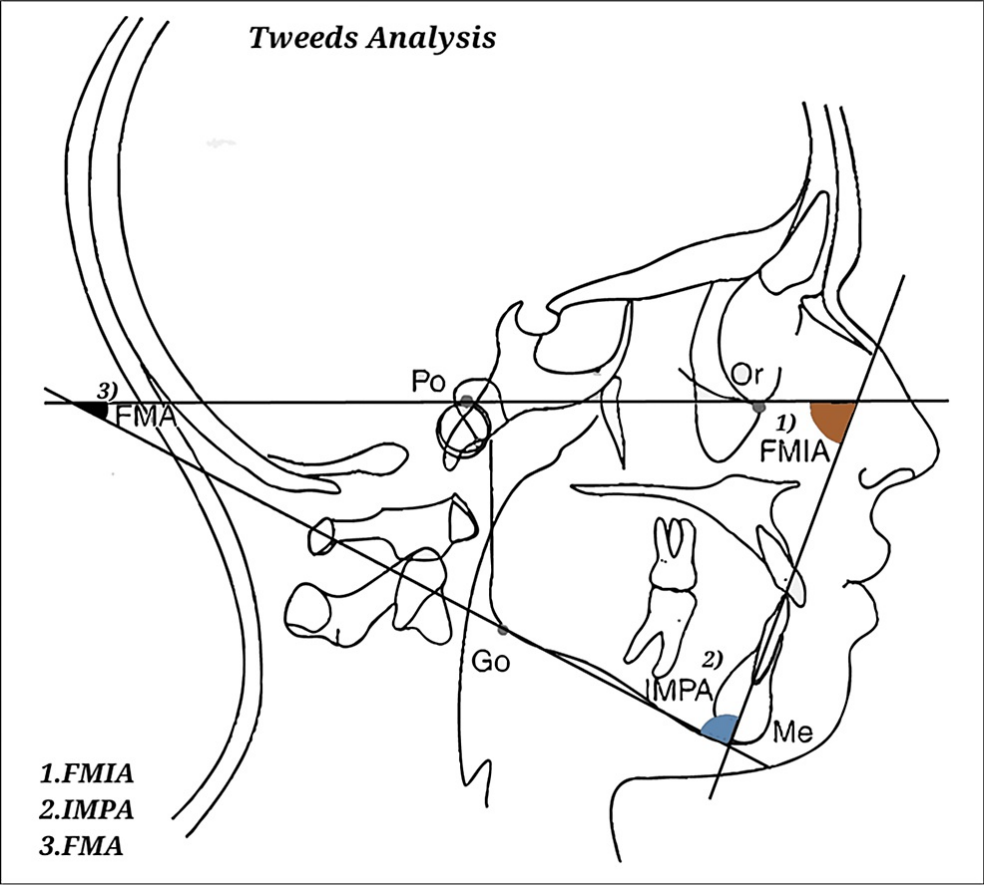
Tweed’s analysis. Image has been created by the authors of this article. FMA, Frankfort-mandibular plane angle; FMIA, Frankfort-mandibular incisor angle; IMPA, incisor mandibular plane angle

Downs’, Steiner's, and Tweed's approaches were employed for a comprehensive cephalometric analysis, as depicted in Figures 1-3.

Image enhancement and standardization

Techniques

Image magnification, contrast enhancement, and Adobe Photoshop (Adobe Systems Incorporated, San Jose, USA) were utilized for resizing cephalometric radiographs to standard lateral head film dimensions (8 x 10 inches).

Calibration

An 8-inch ruler scale was included in the images for basic calibration, and sequential numbering was added for ease of identification.

In this study, digital tracing was done using NemoCeph. However, manual tracing can also be done. Overall, the combination of digital analysis and manual tracing provided a comprehensive evaluation of cephalometric parameters for the study of BCLP individuals.

Manual tracing protocol implementation

Protocol

Manual tracing was identified as a necessary addition to the methodology. Transparent overlays will be used to trace specific anatomical structures and landmarks on the radiographs.

Precision

Tracings will be meticulously executed to ensure accuracy in identifying key points and measurements required for the analysis.

Comparison

Results from manual tracing will be compared with digital measurements to assess consistency and validate findings, enhancing the overall robustness of the study.

Statistical analysis

Methods

Methods involved utilizing Student’s t-test and correlation analysis to identify patterns between individuals with and without CLP conditions.

Analytical Approach

The analytical approach was guided by Philine H. Doberschutz's research, employing an analytical formula referenced from Lachin (1981) for data analysis.

Software

Software utilized for efficient data processing and interpretation was SPSS Version 27.0 (IBM SPSS Statistics for Windows, IBM Corp., Armonk, NY).

This comprehensive methodology ensured systematic data collection, adherence to ethical standards, standardized analysis techniques, and robust statistical analysis to effectively address the research objectives.

Figures 1-3 show the parameters listed in Table 1 on a lateral cephalogram.

**Table 1:** Cephalometric parameters used in the study AB plane angle, A point-B point to nasion-pogonion; ANB, A point-nasion-B point; Lo. 1 to NB, mandibular incisor to nasion-B point; UP 1 to A-pog, maxillary incisor to A point-pogonion; UP 1 to NA, upper incisor to nasion-A point; SNA, sella-nasion-A point; SNB, sella-nasion-B point

Sr. no.	Dental parameters	Skeletal parameters
1	UP 1 to NA	Facial angle
2	UP 1 to NA (mm)	Angle of convexity
3	Lo. 1 to NB	Mandibular plane
4	Lo. 1 to NB (mm)	Y axis
5	Inter-incisal angle	AB plane angle
6	Cant of occlusal plane	SNA
7	Inter-incisal angle	SNB
8	Incisor to occlusal plane angle	ANB
9	UP 1 to A-pog	Occlusal plane angle

The sella, nasion, A point, orbitale, B point, gnathion, gonion, lower incisor incisal edge, pogonion, menton, Frankfort horizontal, maxillary plane, mandibular plane, S-N line, S-A line, S-B line, sella-gonion-menton (S-GoMe) line, A-pog line, A-N line, A-B line, upper incisor to A-pog line angle and lower incisor to A-B line angle were noted as landmarks.

The importance of these cephalometric measurements lies in their ability to offer specific insights into the unique craniofacial characteristics present in this particular group. Each parameter has a distinct meaning:

Sella-nasion-A point angle (SNA): This measurement helps gauge the front-to-back relationship of the upper jaw in relation to the cranial base, shedding light on how the cleft impacts the upper jaw's positioning.

Sella-nasion-B point angle (SNB): SNB provides information about the front-to-back alignment of the lower jaw concerning the cranial base, giving an understanding of how the cleft affects the lower jaw's position.

A point-nasion-B point angle (ANB): This angle highlights the discrepancy between the positions of the upper and lower jaws. Deviations can indicate the extent to which the cleft has affected the anteroposterior relationship of the jaws.

Mandibular plane angle: By measuring the slope of the jawbone in relation to the cranial base, this angle uncovers deviations that affect both the vertical and horizontal orientations of the jaw in cleft patients.

Occlusal plane angle: This angle gauges the tilt of the occlusal plane compared to the cranial base. Variations can impact how the upper and lower teeth align and function, essential aspects in cleft patients.

UP 1 to NA (in millimeters): Assessing the vertical alignment of the upper incisor relative to the nasion offers insights into potential vertical differences associated with the cleft.

Lo. 1 to NB (lower incisor to nasal bone): This measurement evaluates the lower incisor's position relative to the nasal bone, aiding in understanding the alignment of the lower teeth and jaw concerning the upper jaw.

Inter-incisal angle: The inclination of the incisors is quantified through this angle. Perturbations here can influence the position and arrangement of the incisors, which play a pivotal role in proper occlusion and appearance.

AB plane angle: By examining the anteroposterior relationship between the upper and lower jaws in a three-dimensional manner, this angle provides insight into skeletal imbalances influenced by the cleft.

Incisor to occipital plane angle: This angle informs about the inclination of the incisors relative to the occipital plane, giving clues about how the cleft impacts both facial aesthetics and bite alignment.

UP 1 to A-pog (anterior point of palatal plane to A-pogonion): Measuring the vertical position of the upper incisor in relation to the anterior point of the palatal plane and A-pog helps comprehend the vertical dimensions in play.

Y axis: By evaluating the vertical alignment of the upper incisor relative to the cranial base, this parameter contributes to understanding overall facial harmony and vertical relationships affected by the cleft.

Angle of convexity: This angle portrays the degree of convexity in the facial profile. Shifts here indicate how the cleft condition affects the overall facial appearance and skeletal relationships.

## Results

The study identified statistically significant measurements, including SNA, SNB, ANB, mandibular plane angle, occlusal plane angle, UP 1 to NA, UP 1 to NA (in millimeters), Lo. 1 to NB, inter-incisal angle, AB plane angle, Incisor to occipital plane angle, UP 1 to A-pog, Y axis, and angle of convexity. These findings indicated a class III malocclusion, highlighting the need for personalized treatment strategies.

However, measurements such as Lo. 1 to NB (in millimeters), facial angle, Y axis, angle of convexity, mandibular plane angle, and cant of occlusal plane angle did not significantly differ between cleft and non-cleft groups. Nonetheless, the identified class III malocclusion emphasizes the importance of tailored treatment approaches.

A novel approach for approximating A point in bilateral CLP patients using stable landmarks derived from the nasion was introduced, offering potential improvements in cephalometric analysis accuracy, especially in cases with challenging maxillary growth deformities.

Rigorous measures, including acquiring three measurements for each parameter and utilizing NemoCeph software to minimize manual tracing errors, ensured data reliability. For a comprehensive overview of statistically significant findings, refer to Tables 2-4 in the study.

**Table 2:** Comparison of Downs’ parameters in two groups The parameters with significant values include AB plane angle, inter-incisal angle, incisor to occlusal plane angle, and upper incisor to A-pogonion distance. AB plane angle, A point-B point to nasion-pogonion; UP 1 to A-pog, maxillary incisor to A point-pogonion

Parameters	Cleft		Normal		t-value	p-value
	Mean	SD	Mean	SD		
Facial angle	85.36	7.69	84.84	5.25	0.279	0.781, NS
Y axis	60.56	6.51	60.28	10.47	0.114	0.910, NS
Angle of convexity	7.2	5.79	6.04	7.90	0.592	0.557, NS
Mandibular plane angle	23.16	5.96	24.4	6.28	1.721	0.092, NS
Cant of occlusal plane	12.16	5.05	10.96	4.90	0.853	0.398, NS
AB plane angle	7.12	4.46	-1.04	9.25	3.973	0.0001, S*
Inter-incisal angle	149.96	12.59	111.48	11.06	11.001	0.0001, S*
Incisor to occlusal plane angle	15.48	3.66	37.68	21.99	4.978	0.0001, S*
UP 1 to A-pog	-1.12	2.70	8.64	4.22	9.741	0.0001, S*

**Table 3:** Comparison of Steiner’s parameters in two groups The parameters with significant values include SNA, SNB, ANB, mandibular plane angle, occlusal plane angle, upper incisor to nasion angle, upper incisor to nasion distance in millimeters, lower incisor to NB, and inter-incisal angle. Lo. 1 to NB, lower incisor to nasal bone; UP 1 to NA, upper incisor to nasion-A point; UP 1 to NB, upper incisor to nasion-B point

Parameters	Cleft		Normal		p-value
	Mean	SD	Mean	SD	
SNA	81.04	4.55	83.48	3.73	0.710, S*
SNB	83.28	5.80	81.56	4.81	0.259, S*
ANB	1.08	2.47	2.08	2.90	0.195, S*
Mandibular plane	23.16	7.83	27.96	6.19	0.0001, S*
Occlusal plane angle	19.48	5.72	13.56	5.92	0.001, S*
UP 1 to NA	23.88	5.35	32.00	6.66	0.0001, S*
UP 1 to NA (mm)	5.00	0.82	6.20	2.52	0.028, S*
Lo. 1 to NB	23.28	3.51	32.76	8.36	0.0001, S*
Lo. 1 to NB (mm)	4.92	0.81	5.96	2.92	0.093, NS
Inter-incisal angle	149.96	11.66	111.48	11.04	0.0001, S*

**Table 4:** Comparison of Tweed’s parameters in two groups Significant values are with Y axis and angle of convexity for the parameters.

Parameters	Cleft		Normal		p-value
	Mean	SD	Mean	SD	
Facial angle	29.68	6.75	26.24	6.95	0.083, NS
Y axis	81.92	11.45	100.60	9.31	0.0001, S
Angle of convexity	76.16	14.94	52.40	10.24	0.0001, S

**Figure 4:**
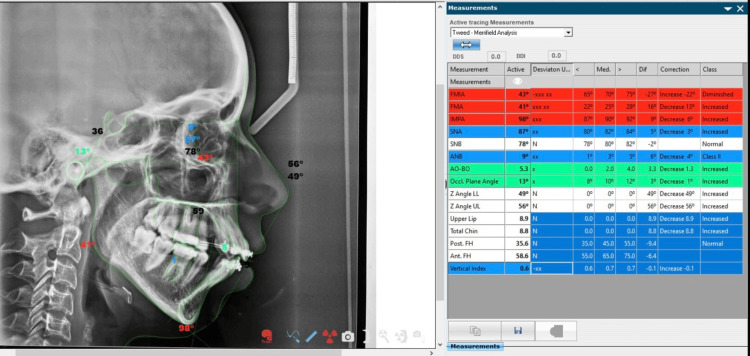
This excerpt is a captured portion of Tweed's analysis extracted from NemoCeph NX version 8.0.

**Figure 5:**
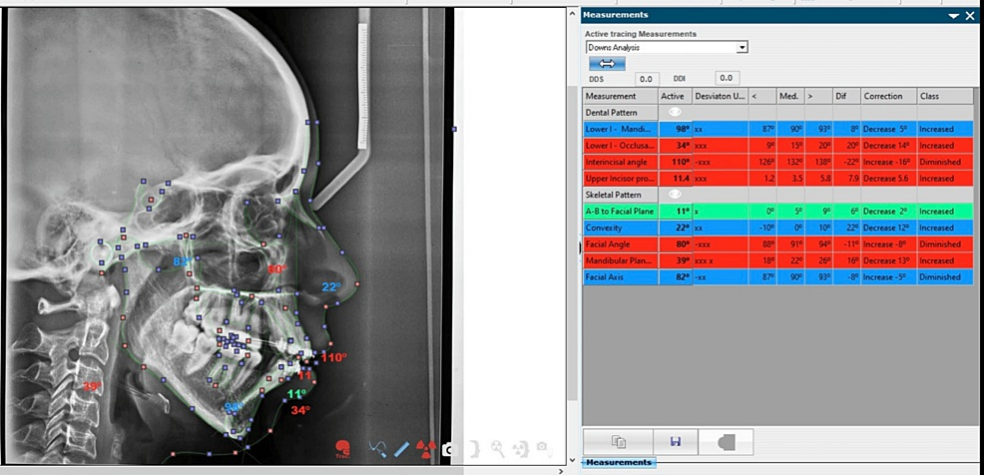
This excerpt is a captured portion of Downs’ analysis extracted from NemoCeph NX version 8.0.

**Figure 6:**
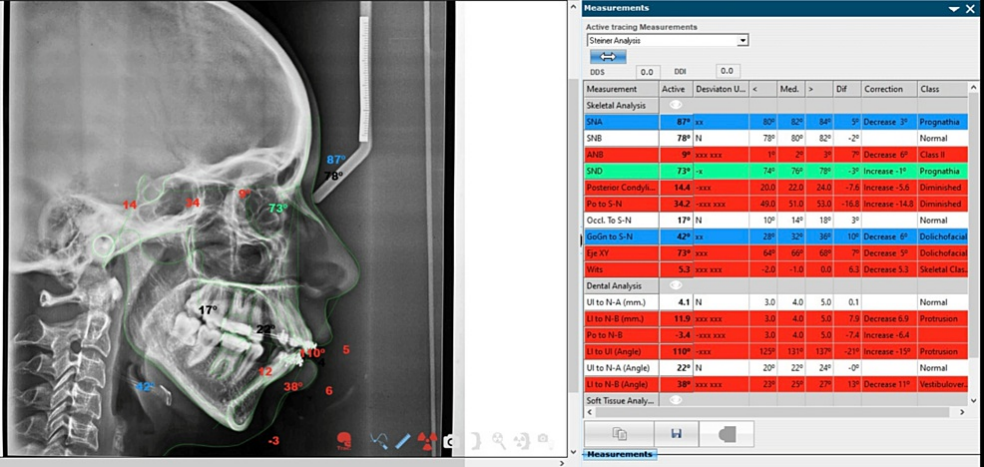
This excerpt is a captured portion of Steiner's analysis extracted from NemoCeph NX version 8.0.

Figures 4-6 show snapshots of the analysis performed in the NemoCeph software.

## Discussion

In the Central India population, a study revealed a prevalence of 0.66% for CLP and 0.27% for cleft palate alone. This study established a strong association between CLP and environmental risk factors like nutritional deficiencies, anemia, and self-administration of medications. The research was prompted by the need to address the scarcity of comprehensive cephalometric data for individuals with non-syndromic BCLP within the Indian community. This is significant due to the unique genetic predispositions present in different populations.

There were fewer comparable cephalometric mean values for BCLP patients in the Indian community, which is significant since distinct genetic predispositions exist in various populations, due to which research was designed to solve this current problem. Cephalometric analysis is a crucial diagnostic technique for creating a treatment strategy. Cephalometric radiography and analysis done in the past required human labor and were error-prone. The technique is likewise riddled with flaws [16]. Due to restrictions on the utility of two-dimensional cephalometry, the world is shifting to a digital mode of intervention.

In a study by Paul et al., compared to manual or semiautomatic tracing (NemoCeph), automated tracing (WebCeph) exhibited more landmark recognition errors. In comparison to manual tracing, WebCeph and NemoCeph both proved to be more reliable than the latter, with NemoCeph exhibiting higher efficacy [17]. Digital cephalometry reduces intraobserver and interobserver variations, giving more accurate data for the study. Due to the underdevelopment of the maxilla in patients with BCLP, it is challenging to locate landmarks for the study [18]. Researchers are concentrating on landmark locations and on-screen tracing due to the rapid improvement of digital radiography. Through computer-assisted cephalometric analysis on digitized cephalograms, this method dramatically lowers the possibility of mistakes, eliminates the need for physical copies, and saves time [19]. NemoCeph software, which has an advantage over conventional tracing methods by minimizing observer mistakes, was employed for this investigation. Due to the anatomical variance in CLP patients, the conventional mean values found in Downs’, Steiner's, and Tweed's study for the normal population cannot be applied. Therefore, research to re-evaluate those aspects for CLP patients in India was necessary. Due to the lack of metrics like anterior nasal spine (ANS), A point, and posterior nasal spine (PNS), these individuals have had analysis issues. According to Diagavane et al., point ANS is positioned approximately 2-5 mm anterior to the perpendicular line N [19]. Its linear distance from the N perpendicular (ANS') line is approximately half that of Ba'-A point [20]. According to Jacobson et al., an acceptable position (approximated as A point) was identified as a point located 3.0 mm toward the outer lip surface from a point situated between the upper third and lower two-thirds of the vertical axis of the maxillary central incisor's root. This particular point also closely resembles the true NA plane [16]. From birth to the age of 20, BCLP patients get therapy. Surgery is the sole option for treating additional defects and the maxillary deficit. An age range of 10 to 18 years was chosen for this study, taking into account the active growth occurring and the surgery they would eventually endure during that time.

The maxillary process is distorted, making it difficult to detect A point and analyze the skeletal and dental characteristics that are essential for understanding the development pattern and skeletal growth in CLP patients. For the purpose of this research, SNA, SNB, and ANB are crucial. With a mean value of 81.04, SNA suggests a recessive site for the maxilla, while SNB's mean value of 83.28 suggests mandibular prognathism. ANB with a mean value of 1.08 indicates class III with mandibular prognathism in relation to the cranial base, indicating maxillary deficit and its treatment, especially in cases involving CLP; surgical intervention gains significance as it determines the pre- and post-surgery position of A point, as well as the degree of orthodontic proclination required for proper incisor placement within the cortical bone's midpoint. The alignment and orientation of teeth hold significance for stability in the premaxillary area. Cephalometric analysis is essential due to the limitations set by a defined range of discrepancies in BCLP cases, which necessitates effective management. Cephalometric analysis is a functional tool for orthodontists by providing benefits in various arenas in dentistry. Burak et al. described the importance of the cephalometric study as that it can help to optimize future treatment protocols, which helps to achieve the greatest potential function and aesthetics to raise the patient's quality of life [21]. Cephalometric analysis plays a crucial role in orthodontics by providing information about the patient's skeletal and dental relationships, aiding in further diagnosis, treatment management, and predicting the outcome of the intervention.

This form of analysis serves a dual purpose, extending its utility beyond diagnostics. It can be harnessed in the realm of aesthetics and smile design, allowing for a comprehensive evaluation of the alignment between facial features and envisioned dental alterations. This assessment encompasses factors such as lip position, chin prominence, and smile symmetry, all of which are integral to achieving an aesthetically pleasing result.

Furthermore, cephalometric analysis provides invaluable insights into the patient's skeletal anatomy. This information is instrumental in enabling surgeons to meticulously plan surgical procedures with a high degree of accuracy. This aspect is particularly crucial in the context of orthognathic surgery, a field dedicated to rectifying severe jaw discrepancies. The ultimate goal of such procedures is to enhance both functional and aesthetic aspects, making precise planning and prediction of outcomes paramount.

Limitations

However, the study has its limitations. It focused exclusively on individuals with BCLP due to the severity of the condition, which resulted in a smaller sample size. Additionally, the intended three-year follow-up with repeated lateral cephalometric radiographs could not be executed as planned. Nevertheless, the study provides valuable insights into the significance of cephalometry in the treatment of CLP patients and the ongoing efforts to enhance its precision and effectiveness.

## Conclusions

In conclusion, this research undertook an extensive comparative examination of the skeletal and dental characteristics of individuals afflicted with BCLP in the Central Indian region. We meticulously scrutinized Downs’, Steiner's, and Tweed's analyses by utilizing lateral cephalograms, shedding light on the unique challenges that the fields of orthodontics and dentistry encounter when dealing with BCLP cases. Our results emphasize the critical importance of customizing cephalometric assessments for BCLP patients, recognizing the diverse genetic influences prevalent in different populations. The inherent anatomical variations and growth restrictions in BCLP anomalies create complexities in identifying and measuring landmarks, rendering conventional mean values from standard population studies unsuitable for direct application. To enhance measurement precision and reduce errors in cephalometric analysis, we harnessed advanced technology, including the NemoCeph software. Our efforts were directed toward resolving issues related to landmarks like A point and others, with the goal of establishing a more accurate framework for the diagnosis, treatment planning, and implementation of orthognathic procedures in BCLP patients. Our comparative analysis unveiled significant disparities in various parameters, encompassing SNA, SNB, ANB, inter-incisal angle, and UP 1 to A-pog. These disparities underscore the pressing necessity of tailoring treatment strategies to meet the unique requirements of each BCLP patient, ultimately ensuring functional and aesthetic success. In summary, our study enriches our comprehension of cephalometric analyses concerning BCLP cases in the Central Indian population. The distinctive challenges posed by BCLP anomalies demand a personalized approach to diagnosis and treatment planning, taking into account the distinctive skeletal and dental characteristics inherent in these patients. Embracing advanced technology and individualized assessments promises to elevate the quality of care and the outcomes achieved for individuals affected by BCLP anomalies.
